# KL-6 levels in the connective tissue disease population: typical values and potential confounders–a retrospective, real-world study

**DOI:** 10.3389/fimmu.2023.1098602

**Published:** 2023-06-20

**Authors:** Aiyuan Zhou, Haiyun Tang, Wenzhong Peng, Yanan Wang, Xiaoping Tang, Hang Yang, Rongli Lu, Pinhua Pan

**Affiliations:** ^1^ Department of Respiratory Medicine, National Key Clinical Specialty, Branch of National Clinical Research Center for Respiratory Disease, Xiangya Hospital, Central South University, Changsha, Hunan, China; ^2^ Center of Respiratory Medicine, Xiangya Hospital, Central South University, Changsha, Hunan, China; ^3^ Department of Respiratory Medicine, Clinical Research Center for Respiratory Diseases in Hunan Province, Changsha, Hunan, China; ^4^ Department of Respiratory Medicine, Hunan Engineering Research Center for Intelligent Diagnosis and Treatment of Respiratory Disease, Changsha, Hunan, China; ^5^ National Clinical Research Center for Geriatric Disorders, Xiangya Hospital, Changsha, Hunan, China; ^6^ Department of Radiology, Xiangya Hospital, Central South University, Changsha, Hunan, China

**Keywords:** CTD, ILD, KL-6, CTD-ILD, CTD-NILD

## Abstract

**Background:**

Krebs von den Lungen 6 (KL-6) is a potential biomarker for determining the severity of interstitial lung disease (ILD) in patients with connective tissue disease (CTD). Whether KL-6 levels can be affected by potential confounders such as underlying CTD patterns, patient-associated demographics, and comorbidities needs further investigation.

**Methods:**

From the database created by Xiangya Hospital, 524 patients with CTD, with or without ILD, were recruited for this retrospective analysis. Recorded data included demographic information, comorbidities, inflammatory biomarkers, autoimmune antibodies, and the KL-6 level at admission. Results of CT and pulmonary function tests were collected one week before or after KL-6 measurements. The percent of predicted diffusing capacity of the lung for carbon monoxide (DLCO%) and computed tomography (CT) scans were used to determine the severity of ILD.

**Results:**

Univariate linear regression analysis showed that BMI, lung cancer, TB, lung infections, underlying CTD type, white blood cell (WBC) counts, neutrophil (Neu) counts, and hemoglobin (Hb) were related to KL-6 levels. Multiple linear regression confirmed that Hb and lung infections could affect KL-6 levels independently; the β were 9.64 and 315.93, and the P values were 0.015 and 0.039, respectively. CTD-ILD patients had higher levels of KL-6 (864.9 vs 463.9, *P* < 0.001) than those without ILD. KL-6 levels were closely correlated to the severity of ILD assessed both by CT and DLCO%. Additionally, we found that KL-6 level was an independent predictive factor for the presence of ILD and further constructed a decision tree model to rapidly determine the risk of developing ILD among CTD patients.

**Conclusion:**

KL-6 is a potential biomarker for gauging the incidence and severity of ILD in CTD patients. To use this typical value of KL-6, however, doctors should take Hb and the presence of lung infections into account.

## 1 Introduction

Connective tissue diseases (CTDs), including systemic lupus erythematosus (SLE), rheumatoid arthritis (RA), systemic sclerosis (SSc), inflammatory myositis (IM), Sjogren’s syndrome (SS), and mixed connective tissue disease (MCTD), are commonly complicated by the involvement of many organ systems, such as the lung and the kidney (1, 2). CTD-related pulmonary lesions are mainly manifested as interstitial changes, which are named CTD-interstitial lung disease (CTD-ILD). CTD-ILD is one of the main causes of high morbidity and even mortality among CTD patients. High-resolution computed tomography (HRCT) is an important means of differentiating early ILD. However, it is challenging to perform routine screening due to the limitations of cost, radiation, and other considerations; as such, the identification of biomarkers able to recognize ILD could decrease economic costs and increase the timeliness of therapy to improve patient outcomes.

It is generally accepted that alveolar epithelial cell (AEC) injury is the key event for the occurrence of ILD. The published studies have shown that damaged AEC can secrete a large number of pro-fibrotic factors, provoking the migration, proliferation, activation, and myofibroblast differentiation of fibroblasts and causing the accumulation of extracellular matrix, leading to irreversible lung fibrosis (3–5). Therefore, measuring the level of molecules secreted by damaged epithelial cells is a potential means to assess the severity of the injury and to predict the incidence of ILD.

Krebs von den Lungen-6 (KL-6) is a circulating high-molecular-weight mucin-like glycoprotein, also categorized as MUC1, that is expressed primarily on alveolar type II pneumocytes and bronchial epithelial cells. Accumulation of KL-6 can further disrupt alveolar capillaries and the regeneration of AEC2 (6, 7). Meanwhile, patients with ILD often also have systemic inflammatory response syndrome, leading to more severe AEC2 damage and more KL-6 release (8). Additionally, KL-6 is reported to be one of the key molecules involved in epithelial-mesenchymal interactions by regulating myofibroblast differentiation (9). Because there are no epitopes in animals other than apes, there have been few animal studies on KL-6. However, KL-6 has been reported to be detectable in mice expressing human MUC1(hMUC1-exp) mice and can reflect the severity of bleomycin-induced lung fibrosis (10). All of the above factors may be the underlying pathological mechanisms of KL-6 to predict the incidence of ILD and to predict its prognosis.

The available studies have shown that elevated serum levels of KL6 are related to disease severity (11–14). However, there are drawbacks to these previous studies: the greatest issue is the exclusion of patients with comorbidities. Patients with CTD were more likely to also have cancer (15, 16), lung infections (17), and tuberculosis(TB) (18, 19). These comorbidities may also result in abnormal KL-6 levels, which may cause some confusion for clinicians attempting to judge the presence or severity of ILD. Additionally, the conclusions are not convincing enough because the sample size is typically low-less than 100 cases. Moreover, KL-6 levels at baseline may vary depending on the different underlying types of CTD. In addition, whether patient-related demographic characteristics such as age, gender, and BMI, among others, can affect KL-6 levels also needs further investigation, and these potential confounders may have an influence on the real value of KL-6 in assessing the presence or severity of ILD among patients with CTD.

As such, we performed this retrospective study to determine the potential confounding factors related to KL-6 levels and to re-identify the role of KL-6 among patients with CTD-ILD after adjusting for confounders. Most importantly, we constructed a decision tree model to predict the incidence of ILD.

## 2 Methods

This research was approved by the local Ethics Committee of the Xiangya Hospital of Central South University and was conducted in accordance with the Declaration of Helsinki and its amendments. The Ethics number is 202104005, and it is approved on May 6^th^, 2021”. The detail information was shown on (https://ethics.tonoinfo.com/#/home/zndxxyyy). Informed consent was waived because of the retrospective nature of the study, and the analysis used anonymous clinical data.

### 2.1 Study design and subjects

This was a retrospective study. Data were collected from the database setup by the Xiangya Hospital of Central South University (Hunan, China). This database recruited patients diagnosed with ILD, COPD, or lung cancer in both the outpatient and inpatient departments of Xiangya Hospital for 20 years. We screened the inpatients with KL-6 measurements in the entire database. For patients with multiple KL-6 tests, we collected the first test on admission. Then, we searched for the discharge diagnosis in the medical records with the following keywords: ‘RA’, ‘SLE’, ‘SSc’, ‘polymyositis (PM)’, ‘dermatomyositis (DM)’, ‘anti-synthetase antibody syndrome’, ‘inflammatory myopathy’, ‘SS’, ‘undetermined CTD’, and ‘mixed CTD’. Then, two pulmonologists and radiologists double checked the CT images of these recruited patients. We excluded patients without CT data. Finally, we divided the recruited patients into a CTD-NILD group and a CTD-ILD group based on the presence of ILD. Professional radiologists and pulmonologists double checked the diagnosis of ILD. The recorded data comprised basic demographic information, including age, gender, BMI, blood type, occupation, smoking history, dust exposure, and atopy history. In addition, regular blood biochemical tests, KL-6 levels, CT scans, and lung function parameters were also collected. All the data were collected at admission. CT and pulmonary function results were collected within 1 week of the KL-6 data.

### 2.2 Pulmonary function data

The pulmonary function test was performed by professional technicians with a spirometer (MasterScreen-Body/Diff, CareFusion, Germany) according to the American Thoracic Society guidelines. The severity of diffusion impairment was assessed by DLCO%, (grade 1, DLCO% ≥80%; grade 2, 60 ≤DLCO% <80%; grade 3, 40 ≤DLCO% <60%, grade 4, DLCO% <40%).

### 2.3 Severity assessment of ILD by CT scan

One professional radiologist, who was blinded to the clinical information, graded the ILD severity of CT scans semi-quantitatively (grade 1, 0–25%; grade 2, 26%–50%; grade 3, 51%–75%; grade 4, 76%–100%) (11).

### 2.4 KL-6 measurements

Serum KL-6 concentrations (µg/mL) were measured through the KL-6 assay using the latex-enhanced immunoturbidimetric assay method by qualified laboratory physicians. 500µg/ml was the cutoff point for differentiating normal and abnormal.

### 2.5 Statistical analysis

Continuous variables are presented as the mean and standard deviation (if the data were normally distributed) and the median and interquartile range (IQR) values (if the data were not normally distributed). Categorical variables are described as frequency rates and percentages. We compared the means for continuous variables with the t-test or analysis of variance (ANOVA) if the data were normally distributed. We used a non-parametric test for non-normally distributed data. We analysed proportions of categorical variables with a χ^2^ test. We used Pearson’s and Spearman’s rank correlation coefficients to analyse the relationship between KL-6 and other parameters. We used a receiver operating characteristic (ROC) curve to determine the cut-off point of KL-6 for predicting the incidence of CTD-ILD. We calculated the results shown in this paper according to the censoring method. We used R Statistical Software (http://www.R-project.org, The R Foundation) and the Free Statistics analysis platform for the statistical analyses. We considered *P* < 0.05 to be statistically significant.

## 3 Results

### 3.1 Clinical characteristics of the study population

**Figure 1:**
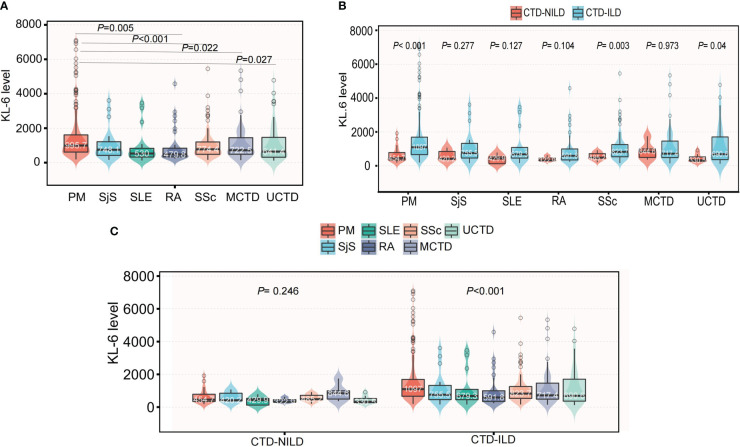
The differences of KL-6 level among specific CTD patterns. **(A)** The differences of KL-6 levels among specific CTD patterns in the whole population. **(B)** Subgroup analysis of the differences of KL-6 level between CTD-NILD and CTD-ILD groups based on underlying CTD pattern. **(C)** Subgroup analysis of the differences of KL-6 level among different CTD patterns based on the presence of ILD.

We retrospectively reviewed 965 inpatients with KL-6 measurements in the database. Overall, 534 patients had a diagnosis of CTD, but we excluded 10 patients due to a lack of CT data. Finally, 524 patients were recruited. Among these patients, 455 were diagnosed with CTD-ILD (**Supplementary Figure 1**). The mean age (55.1 vs 50.9 years, *P* = 0.008) was significantly higher in the ILD group. Other variables, including sex, blood type, the underlying CTD group, and comorbidities, were similar between these two groups. In the CTD-ILD group, there were 31 cases of RA, 13 cases of SLE, 99 cases of SSc, 20 cases of primary SjS, 174 cases of DM, and 65 patients with MCTD and 53 with UCTD (**Table 1**). The lung diffuse function including DLCO (5.0 vs 4.1, *P* = 0.043) and DLCO% (63.4 vs 52.9, *P* = 0.050) were much higher in patients with CTD but without ILD than in patients with CTD-ILD (**Table 2**). Patients with CTD-ILD had lower CRP (7.2 vs 16.1 mg/L, *P* = 0.038), NSE (6.9 vs 8.4 ng/ml, *P* = 0.043) and C4 levels (204.0 vs 241.9 mg/L, *P* = 0.03) than CTD patients without ILD (n = 69). While CTD-ILD patients had higher levels of KL-6 (864.9 vs 463.9 µg/ml, *P* < 0.001) (**Table 2**). When we stratified the data by the type of underlying CTD, we found that PM had the highest KL-6 level (995.7 µg/ml) of all types of CTD (**Figure 1A**). Then, we further analyzed the KL-6 level between CTD and CTD-ILD based on underlying CTD subgroups. We observed that serum KL-6 values were significantly higher in patients with specific CTD-ILD, including PM (1097.0 vs 454.7 µg/ml, *P* < 0.001), SSc (823.7 vs 485.2 µg/ml, *P* = 0.0028), and UCTD (690.6 vs 330.5 µg/ml, *P* = 0.038) (**Figure 1B**). In addition, we found that patients with PM showed the highest KL-6 relative to other CTD patterns in the CTD-ILD group (*P* < 0.001) but presented no difference in the CTD group (*P* = 0.246) (**Figure 1C**).

**Table 1:** Demographic data of all the subjects.

Variables	Total (N=524)	CTD-NILD (N=69)	CTD-ILD (N=455)	*P*
Age(year)	54.5±12.0	50.9±13.1	55.1±11.7	0.008
sex, n (%)				
Female	159 (30.3)	26 (37.7)	133 (29.2)	0.155
Male	365 (69.7)	43 (62.3)	322 (70.8)	
Occupation, n (%)				0.549
Farmers	142 (27.1)	17 (24.6)	125 (27.5)	
Employees	82 (15.6)	12 (17.4)	70 (15.4)	
Freelancer	101 (19.3)	16 (23.2)	85 (18.7)	
Retired	61 (11.6)	7 (10.1)	54 (11.9)	
Unemployed	132 (25.2)	15 (21.7)	117 (25.7)	
Students	6 (1.1)	2 (2.9)	4 (0.9)	
Blood type				0.216
A	51 (9.7)	6 (8.7)	45 (9.9)	
B	35 (6.7)	5 (7.2)	30 (6.6)	
O	63 (12.0)	3 (4.3)	60 (13.2)	
AB	13 (2.5)	1 (1.4)	12 (2.6)	
Unknown	362 (69.1)	54 (78.3)	308 (67.7)	
BMI(kg/m^2^)	22.0 ± 3.2	21.3 ± 3.9	22.1 ± 3.2	0.431
Smoking history, n (%)				< 0.001
No	312 (59.5)	54 (78.3)	258 (56.7)	
Yes	212 (40.5)	15 (21.7)	197 (43.3)	
Occupation exposure, n (%)				0.552
No	473 (97.5)	30 (96.8)	443 (97.6)	
Yes	12 ( 2.5)	1 (3.2)	11 (2.4)	
Specific CTDs				0.289
PM/DM	198 (37.8)	24 (34.8)	174 (38.2)	
SjS	25 ( 4.8)	5 (7.2)	20 (4.4)	
SLE	18 ( 3.4)	5 (7.2)	13 (2.9)	
RA	36 ( 6.9)	5 (7.2)	31 (6.8)	
SSc	116 (22.1)	17 (24.6)	99 (21.8)	
MCTD	70 (13.4)	5 (7.2)	65 (14.3)	
UCTD	61 (11.6)	8 (11.6)	53 (11.6)	
Comorbidities				
Lung cancer	106 (20.2)	11 (15.9)	95 (20.9)	0.341
TB	34 (6.5)	8 (11.6)	26 (5.7)	0.109
COPD	46 (8.8)	3 (4.3)	43 (9.5)	0.163
Severe infections	34 (6.5)	4 (5.8)	30 (6.6)	1
Hospital expense(w)	1.3 (0.9-2.2)	1.3 (0.9-2.2)	1.2 (0.8-2.2)	0.381
Hospital stays	8.0 (7.0-12.0)	8.0 (7.0-11.0)	8.0 (7.0-12.0)	0.868

CTD, connective tissue diseases; CTD-ILD, connective tissue diseases associated interstitial lung disease. BMI, body mass index; RA, rheumatoid arthritis; SLE, systemic lupus erythematosus, SjS, Sjögren’s syndrome; SSc, systemic sclerosis; PM/DM, polymyositis/dermatomyositis; MCTD, mixed connective tissue disease; UCTD, unspecified connective tissue disease.

**Table 2:** Clinical characteristics of the subject population.

Variables	Total	CTD-NILD	CTD-ILD	*P*
Blood routine test				
Hb	116.7 ± 19.9	119.2 ± 24.6	116.4 ± 19.1	0.272
Neu	4.4 (2.9, 6.9)	4.2 (2.9, 7.0)	4.4 (2.9, 6.9)	0.856
Eos	0.1 (0.0, 0.1)	0.1 (0.0, 0.2)	0.1 (0.0, 0.1)	0.866
Lym	1.0 (0.7, 1.4)	1.1 (0.7, 1.6)	1.0 (0.7, 1.4)	0.204
WBC	6.3 (4.4, 9.2)	6.3 (4.4, 9.2)	6.3 (4.4, 9.2)	0.439
Inflammatory marker				
CRP	8.0 (3.3, 22.9)	16.1 (7.4, 25.3)	7.2 (3.2, 22.4)	0.038
ESR	47.0 (18.0, 81.0)	50.0 (0.0, 94.0)	47.0 (21.0, 79.5)	0.678
Autoimmune antibody				
C3	821.0±201.4	846.0 ± 191.4	819.5 ± 202.2	0.558
C4	206.2±77.6	241.9 ± 93.4	204.0 ± 76.2	0.030
dsDNA(+)	17	0	17	1
ANA(+)	228	9	219	0.343
Ro52	126	3	123	0.088
Jo.1	24	0	24	0.610
Scl.70	46	2	44	1
SSB	16	1	15	0.540
SSA	59	2	57	1
KL-6	780.5 (470.4, 1373.0)	463.9 (322.9, 753.9)	864.9 (547.6, 1518.0)	<0.001
Tumor markers				
CEA	1.6 (0.8, 3.1)	1.0(0.8-1.5)	1.7 (0.9, 3.2)	0.073
NSE	7.0 (5.3, 9.0)	8.4 (7.6, 10.7)	6.9 (5.2, 9.0)	0.043
AFP	1.8 (1.4, 2.6)	1.7 (1.4, 3.1)	1.8 (1.4, 2.6)	0.986
CA125	12.6 (7.6, 25.1)	11.1 (9.7, 22.7)	12.6 (7.5, 25.1)	0.785
CA242	4.5 (3.3, 5.4)	5.4 (5.4, 5.4)	4.5 (3.3, 5.4)	0.527
CA199	8.4 (4.6, 17.1)	8.9 (6.1, 11.9)	8.3 (4.6, 17.6)	0.791
Lung function				
FEV_1_	1.9 ± 0.4	2.0 ± 0.3	1.9 ± 0.5	0.082
FEV_1_%	80.9 ± 14.7	83.5 ± 10.2	80.6 ± 15.1	0.186
FVC%	81.9 ± 15.1	84.9 ± 11.6	81.6 ± 15.4	0.132
FVC	2.4 ± 0.6	2.5 ± 0.3	2.4 ± 0.6	0.080
DLCO	4.1 ± 1.4	5.0 ± 2.4	4.1 ± 1.4	0.043
DLCO%	53.4 ± 16.5	63.4 ± 26.7	52.9 ± 15.9	0.050

CTD, connective tissue diseases; CTD-ILD, connective tissue diseases associated interstitial lung disease; Hb, hemoglobulin; Neu, neutrophil; Eos, eosinophil; Lym, lymphocyte; WBC, white blood cell; CRP, C-reaction protein; C3, complement C3; C4, complement C4; ESR, erythrocyte sedimentation rate; KL-6, Krebs Von den Lungen-6; CEA, carcinoembryonic antigen; NSE, neuron-specific enolase; AFP, alpha fetoprotein; CA125,Carbohydrate antigen 125; CA242, Carbohydrate antigen 242; CA199, Carbohydrate antigen 199; FEV_1_, forced expired volume in one second; FVC, forced vital capacity; DLCO, diffusing capacity of the lungs for carbon monoxide.

### 3.2 Factors associated with KL-6 levels

Univariate linear regression analysis showed that BMI, lung cancer, TB, pulmonary infections, white blood cell (WBC) counts, hemoglobin (Hb), neutrophil counts and underlying CTD type were related to the KL-6 level. Age, gender, smoking index, atopy history, dust exposure, and combination with COPD had no effect on the KL-6 level. Patients with high Hb were more likely to present higher KL-6 levels (β = 6.03, *P* = 0.017). Subjects diagnosed with pulmonary infections had higher KL-6 levels than those with only CTD-ILD but no infections. Multiple linear regression confirmed that Hb and pulmonary infections could affect the KL-6 level independently; the β were 9.64 and 315.93, and the *P* values were 0.015 and 0.039, respectively (**Table 3**). The formula for correcting KL6 is Y=-422.24+315.93*1 (when lung infection exists) +9.64 *Hb.

**Table 3:** Factors associated with KL-6 level.

Variables	β	95%CI	*P*	*Adjusted* β	*Adjusted P*
Age	5.13	-3.09~13.35	0.221		
Gender(male)	58.18	-156.44~272.79	0.595		
BMI	39.63	2.67~76.6	0.036	28.61	0.158
Smoking history(No)	104.1	-96.79~304.98	0.309		
Atopy history	53.98	-273.71~381.66	0.746		
Dust exposure	-132.46	-809.16~544.23	0.701		
Comorbidities					
Lung cancer	-335.95	-579.93~-91.97	0.007	-182.91	0.199
TB	-454.06	-852.82~-55.3	0.026	-383.57	0.102
COPD	-150.77	-499.29~197.75	0.396		
Lung infections	331.95	110.73~ 553.16	0.003	315.93	0.039
CTD(ref:PM)					
SjS	-446.85	-918.57~24.86	0.064	-125.39	0.683
SLE	-540.4	-1087.53~6.73	0.053	-209.14	0.589
RA	-576.24	-978.91~-173.56	0.005	-164.11	0.514
SSc	-467.32	-727.18~-207.46	<0.001	-91.15	0.581
MCTD	-362.76	-671.8~-53.72	0.022	-101.93	0.589
UCTD	-369.02	-694.47~-43.57	0.027	0.97	0.996
Blood routine test					
WBC	31.60	6.47~ 56.74	0.014	-124.62	0.165
Eos	-132.68	-803.61~538.24	0.698		
Hb	6.03	1.1~10.97	0.017	9.64	0.015
Neu	39.26	12.43~66.10	0.004	140.32	0.143
Inflammatory marker					
CRP	0.98	-1.49~3.45	0.436		
ESR	1.05	-1.72,3.81	0.457		

BMI, body mass index; TB, tuberculosis; COPD, chronic obstructive pulmonary disease; CTD, connective tissue diseases; RA, rheumatoid arthritis; SLE, systemic lupus erythematosus, SjS, Sjögren’s syndrome; SSc, systemic sclerosis; PM/DM, polymyositis/dermatomyositis; MCTD, mixed connective tissue disease; UCTD, unspecified connective tissue disease; Hb, hemoglobulin; Neu, neutrophil; Eos, eosinophil; WBC, white blood cell; CRP, C-reaction protein; ESR, erythrocyte sedimentation rate; KL-6, Krebs Von den Lungen-6.

### 3.3 The role of KL-6 in assessing the severity of CTD-ILD

**Figure 2:**
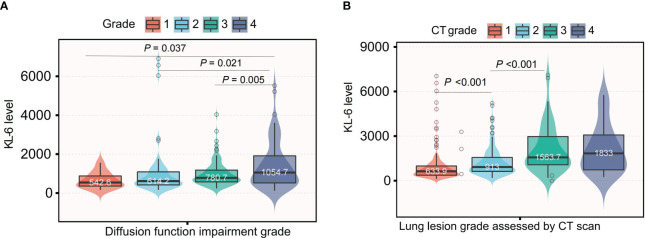
Comparison of KL-6 level based on the severity of ILD assessed by both CT scan and DLCO%; **(A)** Comparison of KL-6 level based on the severity of ILD assessed by DLCO%; **(B)** Comparison of KL-6 level based on the severity of ILD assessed by CT scan.

**Figure 3:**
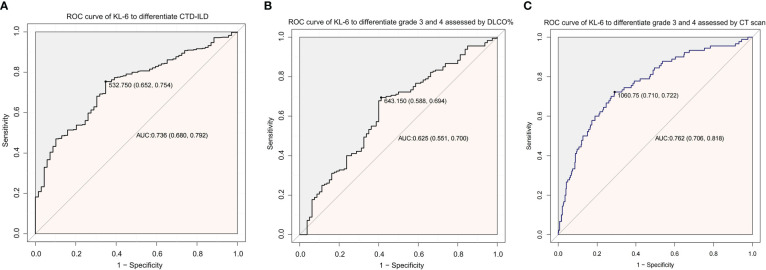
ROC curve analysis to utilize the role of KL-6 in CTD patients. **(A)** ROC curve of KL-6 levels to differentiate the presence of ILD among CTD patients. **(B)** ROC curve of KL-6 levels to differentiate Grade 3 and Grade 4 assessed by DLCO%. **(C)** ROC curve of KL-6 levels to differentiate Grade 3 and Grade 4 assessed by CT scan.

Patients with severe (grade 4) diffusion function impairment presented the highest KL-6 level of all three grades; the mean values of KL-6 in different diffusion impairment grades were 1054.7, 780.7, 614.2, and 542.6(µg/ml), respectively (**Figure 2A**). Additionally, semiquantitative grades of ILD on the CT scan were significantly correlated to the KL-6 level (Rho = 0.426, *P <* 0.001). Serum KL-6 levels successfully differentiated grades 1 and 2 (*P* < 0.001), as well as grades 2 and 3 (*P* < 0.001) in patients with CTD-ILD (**Figure 2B**). Grade 3 and grade 4 showed similar KL-6 levels. Therefore, serum KL-6 can be used to reflect the current status of CTD-ILD defined by CT scans. To utilize the serum KL-6 level in clinical practice, the cut-off points of KL-6 values to predict the presence of ILD in CTD patients were analyzed by ROC. The analysis showed that the KL-6 level at 532.75 U/mL was the best cut-off point for differentiating ILD among CTD patients. The AUC value was 0.736, with a 95% CI of 0.680–0.792. The sensitivity and specificity were 75.4% and 65.2%, respectively (**Figure 3A**). Then, we further explored the cut-off point of KL-6 to assess the severity of ILD among patients with CTD-ILD quantified by semiquantitative grades on CT scans and lung function stratified by DLCO%. The cut-off point for KL-6 was 643.15 µg/ml, the AUC values of KL-6 levels to differentiate grade 3 and 4 defined by DLCO% was 0.625, and the sensitivity and specificity were 69.4% and 58.5%, respectively. In addition, the positive and negative predictive values were 79.1% and 46.8%, respectively (**Figure 3B**). Furthermore, the AUC values of KL-6 levels to differentiate grade 3 and 4 defined by CT scan were 0.762 (95% CI: 0.706–0.818), the negative predictive value was 92.0%, and the cut-off point of KL-6 was 1060.75 µg/ml (**Figure 3C**).

### 3.4 The relationship between KL-6 and lung function, CT scan hints

The KL-6 level showed a mild, negative relationship with FEV_1_%, FVC%, DLCO, and DLCO%. The R values were –0.18, –0.21, –0.20, and –0.24, respectively (**Supplementary Figure 2**).Then, we compared the predictive value of KL6 combined with DLCO and KL6 combined with FVC with that of KL-6 alone to diagnose ILD. We found that KL6, whether combined with DLCO or FVC, had a similar predictive value to ILD as KL-6 alone to diagnose ILD (**Supplementary Figure 3**).

We analysed the association between different CT signs with KL-6 levels and found that patients with CT signs related to ILD, – including ground-glass opacity, honeycomb, and reticular shadow – presented higher levels of KL-6 than patients without these corresponding signs(918.3 vs 703.3µg/ml, *P*=0.003). Other signs, such as emphysema, consolidation, nodules, or tumous, had no relation to the KL-6 level. After adjustment for significant signs, we found that only ILD-related signs had an effect on the KL-6 level β = 415.26, *P* = 0.001 (**Supplementary Figure 4** and **Supplementary Table 1**).

### 3.5 Serum KL-6 levels are associated with the presence of ILD

**Figure 4:**
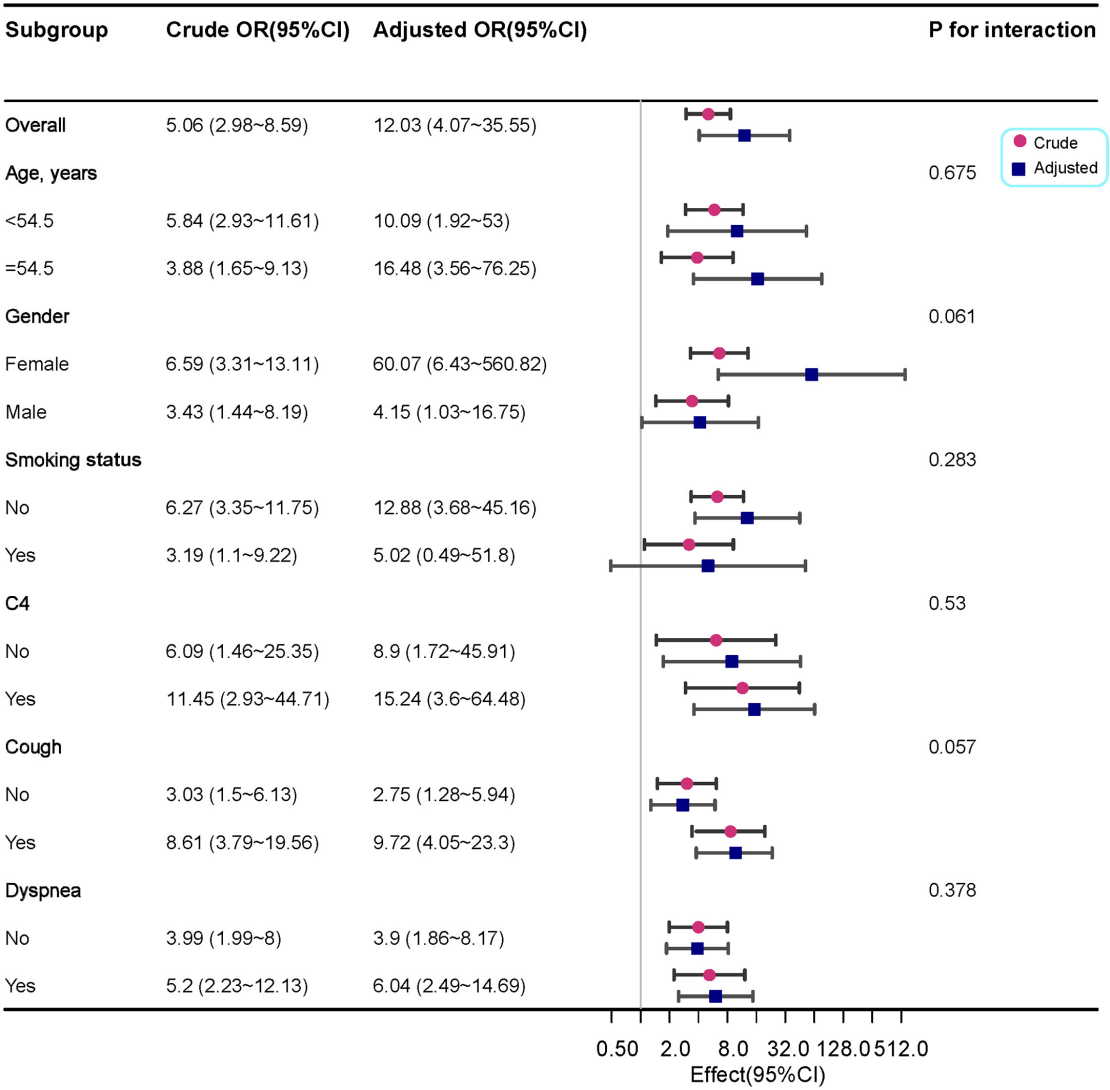
Forest plot for the subgroup analysis of the presence of ILD according to KL-6 levels. For each group of interest, the gray horizontal lines represent the 95% confidence interval (CI).

We performed a logistic regression analysis of factors related to ILD and found that age, cough, dyspnoea, complement C4, smoking status and KL-6 may be related to ILD. Multivariable regression analysis revealed that KL-6 levels were independently associated with ILD (**Table 4**). The likelihood of ILD incidence increased 10.51 times in patients with abnormal levels compared with patients with normal KL-6 levels (OR 10.51, 95%CI 3.7~29.84, *P <*0.001; **Table 4**).To further analyse the stability of the adjusted model, we stratified patients by gender, age, smoking status, cough, dyspnoea, and complement C4. The forest plot revealed that there were no significant interactions between the aforementioned subgroups(*P >*0.05, **Figure 4**).

**Table 4:** Factors associated with ILD.

Variables	OR (95%CI)	*P*	*Adjusted* OR (95%CI)	*P*
Age	2.17 (1.28~3.68)	0.004	0.73 (0.28~1.93)	0.528
Gender(male)	0.68 (0.4~1.16)	0.157		
Occupation	0.79 (0.36~1.76)	0.568		
Smoking Status	2.75 (1.51~5.02)	0.001	3.81 (1.22~11.85)	0.021
Atopy history	0.47 (0.18~1.22)	0.12		
Dust exposure	0.74 (0.09~5.96)	0.781		
Comorbidities				
Lung cancer	1.39 (0.7~2.75)	0.343		
TB	0.46 (0.2~1.07)	0.071		
COPD	2.3 (0.69~7.62)	0.174		
Lung infections	1.65 (0.87~3.12)	0.124		
CTD (ref:PM)				
SjS	0.55 (0.19~1.61)	0.275		
SLE	0.36 (0.12~1.09)	0.072		
RA	0.86 (0.3~2.41)	0.767		
SSc	0.8 (0.41~1.57)	0.521		
MCTD	1.79 (0.66~4.9)	0.255		
UCTD	0.91 (0.39~2.15)	0.837		
Cough	1.74 (1.05~2.9)	0.033	0.66 (0.22~1.99)	0.458
Dyspnea	2.51 (1.49~4.25)	0.001	0.83 (0.28~2.52)	0.748
Blood test				
WBC	0.98 (0.92~1.04)	0.557		
Eos	0.43 (0.09~1.99)	0.279		
Hb	0.99 (0.98~1.01)	0.272		
Neu	1 (0.93~1.07)	0.954		
CRP	1 (0.99~1)	0.448		
ESR	1 (0.99~1.01)	0.944		
C3	0.94 (0.75~1.17)	0.557		
C4	0.57 (0.34~0.95)	0.032	0.53 (0.3~0.93)	0.027
TC	1.28 (0.88~1.87)	0.193		
LDL	1.34 (0.79~2.3)	0.282		
KL-6	5.06 (2.98~8.59)	<0.001	10.51 (3.7~29.84)	<0.001

ILD, interstitial lung disease; COPD, chronic obstructive pulmonary disease; PM/DM, polymyositis/dermatomyositis; SLE, systemic lupus erythematosus; RA, rheumatoid arthritis; SjS, Sjögren’s syndrome; SSc, systemic sclerosis; MCTD, mixed connective tissue disease; UCTD, unspecified connective tissue disease; WBC, white blood cell; Eos, eosinophil; Hb, hemoglobulin; Neu, neutrophil; CRP, C-reaction protein; ESR, erythrocyte sedimentation rate; C3, complement C3; C4, complement C4; TC, total cholesterol; LDL, Low-density lipoprotein; KL-6, Krebs Von den Lungen-6.

### 3.6 A decision tree based on KL-6 levels to assess ILD

**Figure 5:**
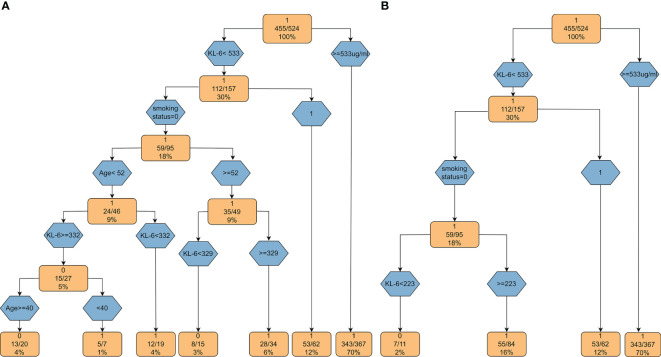
Decision tree models based on KL-6. **(A)** The decision tree model based on the factors obtained by univariate regression analysis; **(B)** The decision tree model based on the variables from multiple regression analysis.

Based on the above factors obtained by logistic regression analysis, we constructed decision tree models (**Figure 5**). When the KL-6 level is > 533 µg/ml, the probability of the patient having ILD is 70%. If the KL-6 level is < 233 µg/ml and there is no smoking history, then the probability of patients having ILD is only 2%. Based on the decision tree, KL-6 is an important factor for evaluating ILD. In the crude model, the weight of KL-6 is 61.9%. After matching various confounding factors, the weight of KL-6 is 75.4%, as shown in **Supplementary Table 2**.

## 4 Discussion

In this study, we comprehensively analysed the factors affecting KL-6 and investigated its clinical significance among patients with CTD. We found that Hb and lung infections could affect the KL-6 level independently. KL-6 presented a significant correlation with the severity of CTD-ILD not only measured with CT scan but also stratified by DLCO%. More importantly, we constructed a decision tree model to determine the presence of ILD, which would be beneficial for future clinical work. To our knowledge, this is the first study to systematically investigate the factors affecting KL-6 levels; additionally, we developed a decision tree model to determine the presence of ILD among patients with CTD in the real world without the exclusion of patients with comorbidities.

KL-6 as a biomarker of lung epithelial cell injury has been widely used among ILD patients. However, whether patients’ self-reported characteristics, including age, BMI, and gender, would have an effect on KL-6 levels remains seldom investigated. In addition, some of the patients have more than one pulmonary disease; as ILD is likely to be combined with lung cancer, COPD, and TB, patients with these diseases would have abnormal levels of KL-6, and most of them were excluded in clinical trials (15, 18, 20), the clinical use of KL-6 levels among these overlap patients should be comprehensively assessed. In this study, we found that Hb and lung infections could affect KL-6 levels independently, suggesting that KL-6 should be better adjusted by Hb and further corrected if patients were complicated by lung infections. The relationship between KL-6 and Hb is seldom reported; a recent study showed that KL-6 and Hb can both be used to assess bone marrow fibrosis (21), but the interaction between KL-6 and Hb is still unknown, and the mechanism through which Hb is correlated to KL-6 needs further investigation. Several studies have shown that lung infections could elevate the expression of KL-6 (22–24). In this study, we confirmed that lung infections could increase the serum KL-6 level after adjusting for other confounders, indicating that clinicians should check CTD patients for anemia or accompanying lung infections once they receive the results of KL-6 levels.

KL-6 has been reported to have a role in evaluating ILD severity among CTD patients. We found that SSc or IM had a relatively high prevalence of ILD compared with RA, SLE, and SjS, which was in close agreement with another study (11), indicating that patients who tend to have SSc, IM, or PM should be screened for ILD more frequently. Patients with PM showed the highest KL-6 relative to other CTD patterns in the CTD-ILD group but presented no difference in the CTD group, suggesting that the underlying CTD type had no effect on the KL-6 level, but patients with PM were more likely to have worse lung conditions.

In previous studies, researchers have reported significant inverse correlations between serum KL-6 levels and DLCO% in patients with polymyositis and dermatomyositis (25, 26). We also found that KL-6 presented a significant correlation with the severity of CTD-ILD not only measured with CT, but also stratified by DLCO%. We further analysed the relationship between CT signs and the KL-6 level and found that the KL-6 level was correlated with ILD signs, including ground-glass opacity, honeycomb, and reticular shadow, but was not related to COPD signs. Doishita et al. (27) also reported positive correlations between KL-6 levels and both the presence and activity of ILD. KL-6, a mucin-associated glycoprotein, may be a trigger for transforming growth factor beta(TGF-β) signalling and fibrosis (28, 29), giving it the potential to be a biomarker not only of the presence of ILD but also of disease activity. A higher KL-6 level indicates greater ground-glass opacity, honeycomb, or reticular shadow lesions, suggesting that KL-6 should be an alternative method to screen ILD among patients with CTD without the radiology-associated risk. More importantly, it may also have the potential to predict the prognosis of patients with CTD-ILD. Multiple logistic regression analysis revealed that KL-6 is an independent predictive factor for the presence of ILD among patients with CTD. Based on this result, we constructed a decision tree model to predict the possibility of ILD based on a set of decision rules.

There are some limitations to the current study. First, we collected the data from our database, patients who had KL6 measurements were more likely to have ILD. Therefore, the sample size of patients with CTD-NILD was much smaller than that of patients with CTD-ILD, but this did not affect our main findings that KL-6 levels should be corrected for Hb and lung infections. Second, not all the patients underwent high-resolution CT. However, in our hospital the scanning thickness is 1.0 mm for conventional CT and 0.6 mm for high-resolution CT, both of which can identify interstitial lesions. Hence, the absence of high-resolution CT would not affect the identification of ILD lesions. Additionally, a small number of patients in CTD-NILD group do not have lung fuction data, but the correlation analysis between KL-6 and the severity of ILD were from the CTD-ILD group, the absence of lung function in the CTD-NILD group had no effect on the main findings. Finally, due to the small sample size, we could not verify the accuracy of the decision tree. Thus, longitudinal cohort studies will be essential in the future.

## 5 Conclusion

Hb and lung infections are independent factors affecting KL-6 levels among CTD-ILD patients. KL-6 is a potential biomarker to predict ILD and assess the ILD severity in the real world.

## Data availability statement

The raw data supporting the conclusions of this article will be made available by the authors, without undue reservation.

## Ethics statement

The studies involving human participants were reviewed and approved by the ethics committee of Xiangya Hospital, Central South University (No. 202104005). Written informed consent for participation was not required for this study in accordance with the national legislation and the institutional requirements.

## Author contributions

AZ, RL, and PP designed the study. All authors contributed toward recruiting subjects, statistical analysis and drafting the paper, and each of the authors agreed to be accountable for all aspects of the work. All authors contributed to the article and approved the submitted version.
