# Supplementary material for: KL-6 levels in the connective tissue disease population: typical values and potential confounders–a retrospective, real-world study
Source: Front Immunol. 2023 Jun 20;14:1098602. doi: 10.3389/fimmu.2023.1098602 (PMC10318146; doi:10.3389/fimmu.2023.1098602)
Supplement: Supplementary file 1 [file Table_1.docx]

Supplement table 1. CT hints associated with KL-6 level

| Variables | β | 95%CI | *P* | *Adjusted* β | *Adjusted P* |
| --- | --- | --- | --- | --- | --- |
| ILD related CT hints | 317.13 | 120.86~513.4 | 0.002 | 415.26 | 0.003 |
| Nodule or tumor | -151.45 | -390.86~87.96 | 0.216 | -203.92 | 0.101 |
| Emphysema | -30.96 | -457.99~396.07 | 0.887 | -35.08 | 0.872 |
| Lung consolidation | 20.17 | -538.25~578.59 | 0.944 | 50.59 | 0.858 |
