# Supplementary material for: KL-6 levels in the connective tissue disease population: typical values and potential confounders–a retrospective, real-world study
Source: Front Immunol. 2023 Jun 20;14:1098602. doi: 10.3389/fimmu.2023.1098602 (PMC10318146; doi:10.3389/fimmu.2023.1098602)
Supplement: Supplementary file 2 [file Table_2.docx]

Supplementary table 2. Weight of each factor in the decision model

| Variables | Crude importance | *Adjusted importance* |
| --- | --- | --- |
| KL-6 | 61.9 | 75.4 |
| Age | 18.3 | 0 |
| Smoking Status | 17.3 | 24.6 |
| Dypnea | 2.3 | 0 |
| Cough | 0.2 | 0 |
| C4 | 0 | 0 |
