# Supplementary material for: KL-6 levels in the connective tissue disease population: typical values and potential confounders–a retrospective, real-world study
Source: Front Immunol. 2023 Jun 20;14:1098602. doi: 10.3389/fimmu.2023.1098602 (PMC10318146; doi:10.3389/fimmu.2023.1098602)
Supplement: Supplementary file 3 [file DataSheet_1.docx]

Supplement Figures


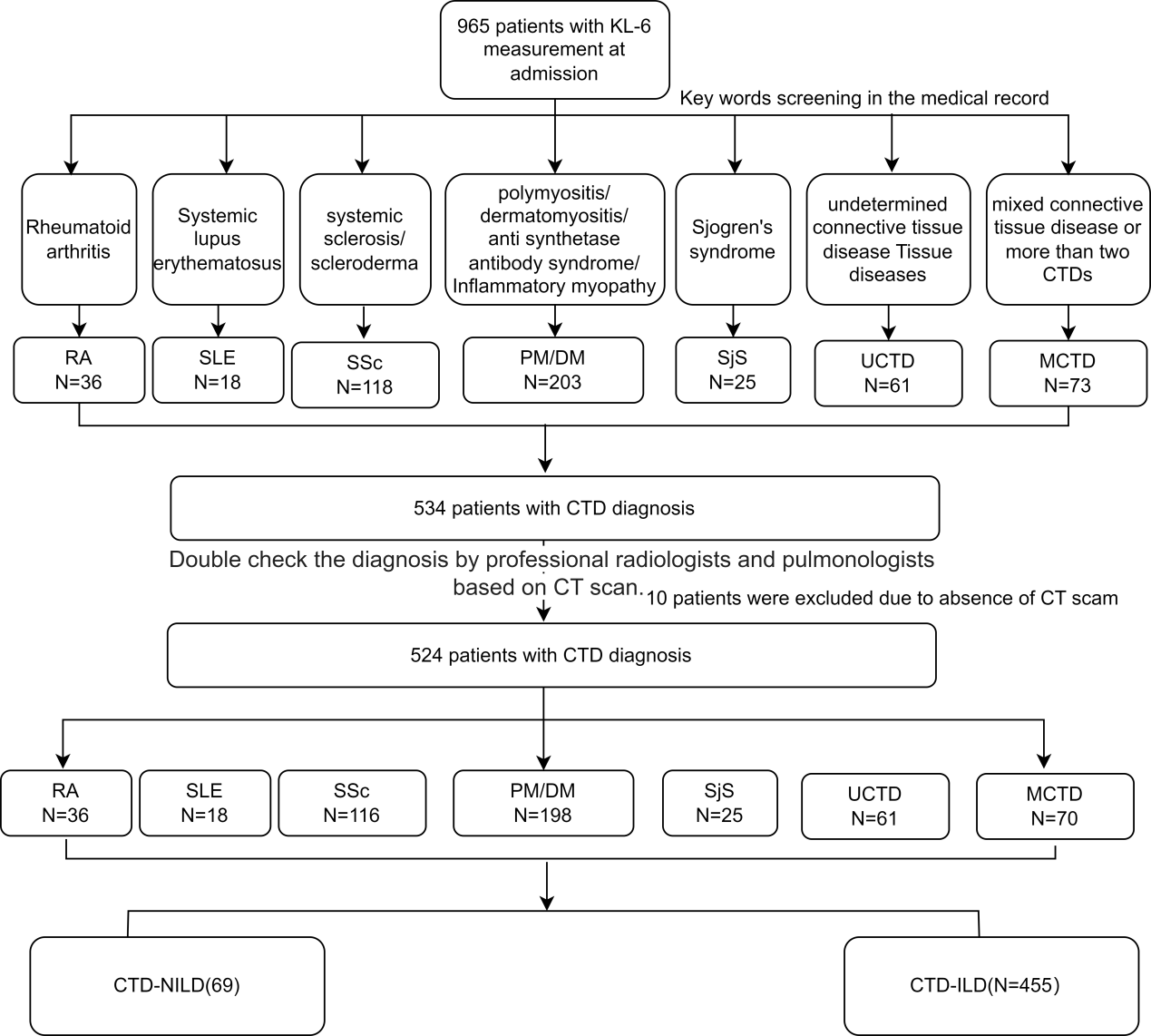


Supplement Figure 1: Flow chart of recruiting patients


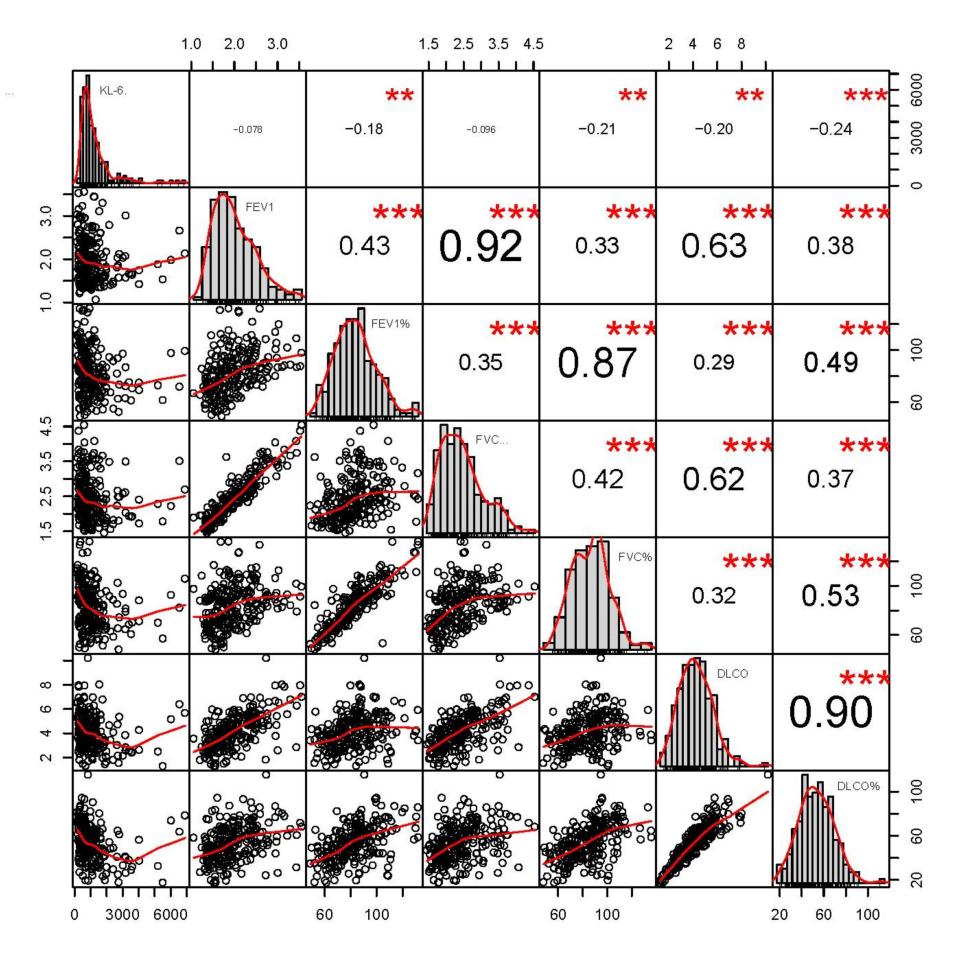


Supplement Figure 2. Association analysis between KL-6 and pulmonary function parameters including FEV1, FEV1%, FVC, FVC%, DLCO and DLCO%.


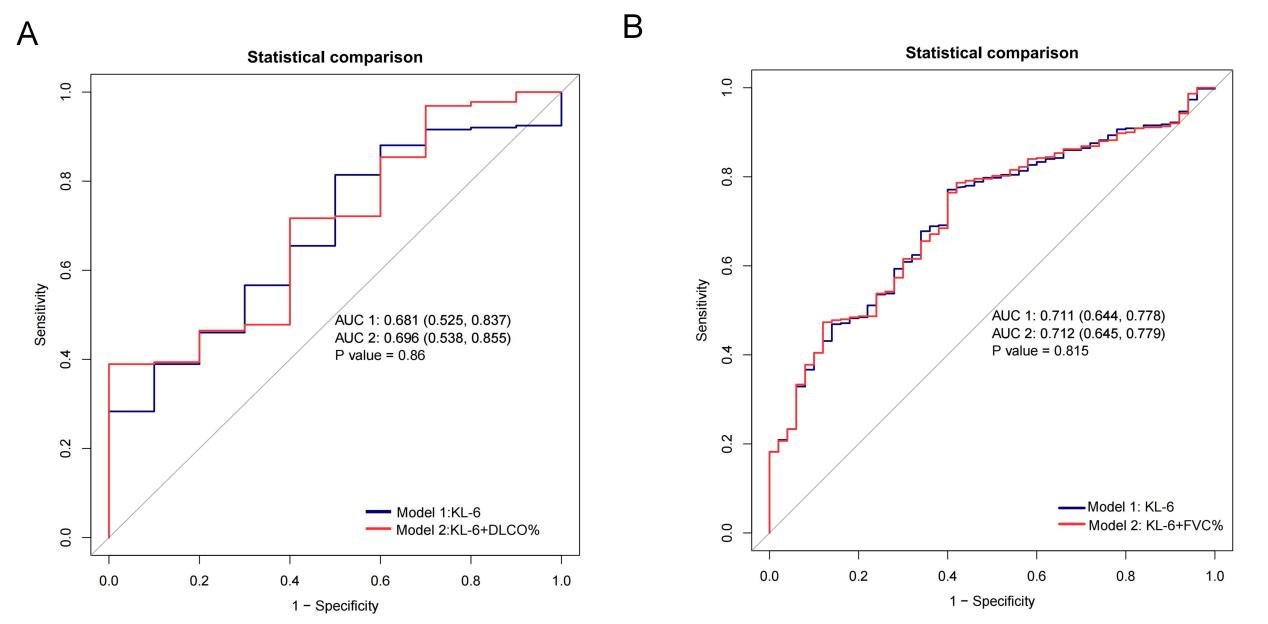


Supplement Figure 3. ROC comparisons of KL-6 and KL-6 combined with lung function(DLCO% and FVC%) to predict the presence of ILD.(A) ROC comparison of KL-6 alone and KL-6 combined with DLCO%;(B)ROC comparison of KL-6 alone and KL-6 combined with FVC%


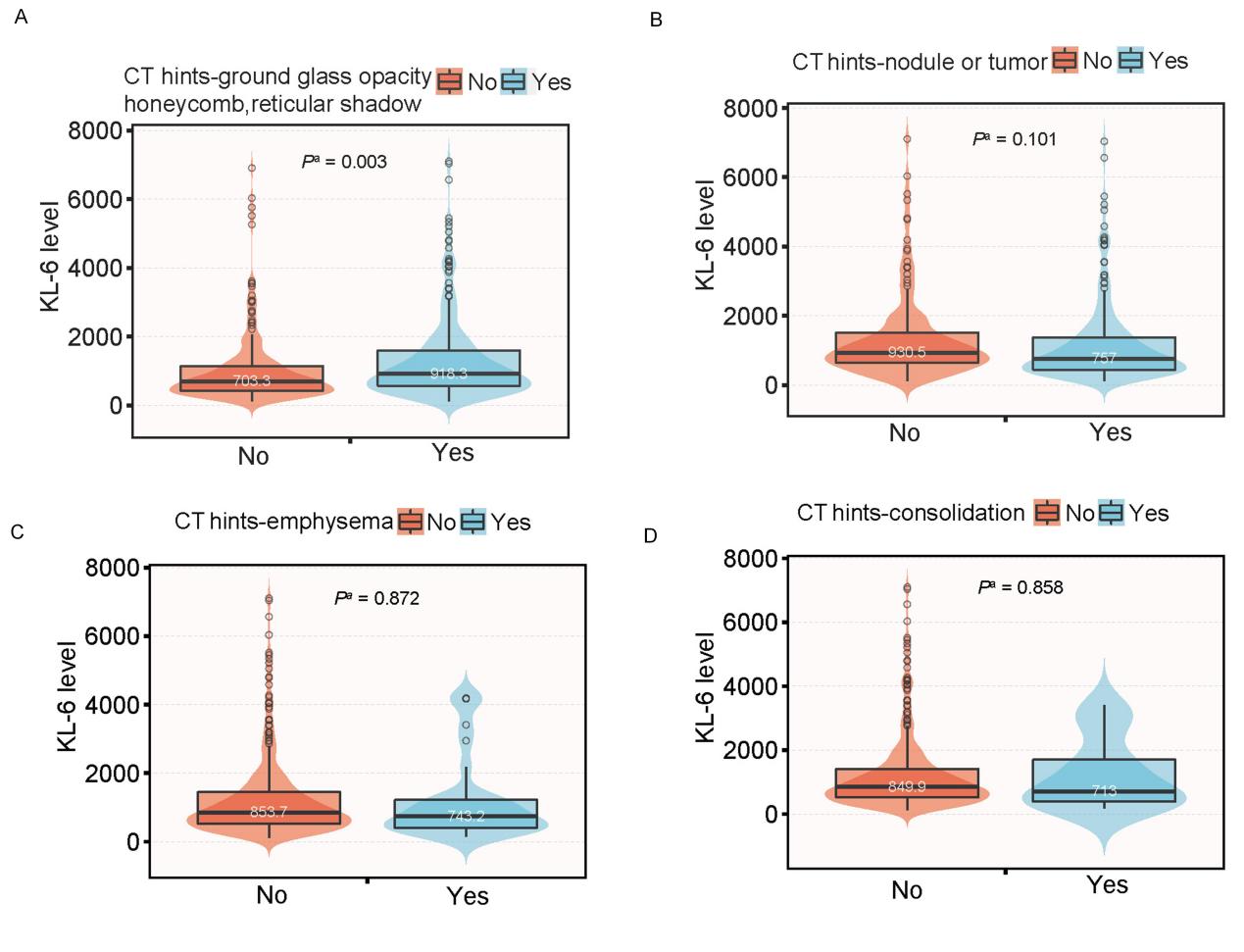
Supplement Figure 4. Comparison of KL-6 level between different CT hints. A. Comparison of KL-6 level between patients with or without ILD related hints; B.Comparison of level between patients with or without nodules; C. Comparison of level between patients with or without emphysema; D. Comparison of level between patients with or without consolidation.
